# Receipt of Breast Milk by Gestational Age — United States, 2017

**DOI:** 10.15585/mmwr.mm6822a1

**Published:** 2019-06-07

**Authors:** Katelyn V. Chiang, Andrea J. Sharma, Jennifer M. Nelson, Christine K. Olson, Cria G. Perrine

**Affiliations:** ^1^Division of Nutrition, Physical Activity, and Obesity, National Center for Chronic Disease Prevention and Health Promotion, CDC; ^2^Oak Ridge Institute for Science and Education, Oak Ridge, Tennessee; ^3^Division of Reproductive Health, National Center for Chronic Disease Prevention and Health Promotion, CDC.

Breast milk is the optimal source of infant nutrition. For the nearly one in 10 infants born prematurely in the United States annually (*1*), breast milk is especially beneficial, helping prevent sepsis and necrotizing enterocolitis and promoting neurologic development (*2*). National estimates of newborn feeding practices by gestational age have not been available previously. CDC analyzed 2017 birth certificate data from 48 states and the District of Columbia (3,194,873; 82.7% of all births) to describe receipt of breast milk among extremely preterm (20–27 weeks), early preterm (28–33 weeks), late preterm (34–36 weeks), and term (≥37 weeks) infants with further stratification by maternal and infant characteristics. The prevalence of infants receiving any breast milk was 83.9% overall and varied by gestational age, with 71.3% of extremely preterm infants, 76.0% of early preterm infants, 77.3% of late preterm infants, and 84.6% of term infants receiving any breast milk. Disparities in receipt of breast milk by several sociodemographic factors, including maternal race/ethnicity, were noted across gestational age groups. These estimates suggest that many infants, particularly infants at high risk for medical complications, might not be receiving breast milk. Efforts are needed to increase the implementation of existing evidence-based policies and practices that support breast milk feeding, particularly for medically fragile infants (*2*,*3*).

The National Vital Statistics System birth data are a census of all live births in the United States. Federal guidance includes procedures for collecting uniform birth data using the U.S. Standard Certificate of Live Birth (*4*).* Data collected include nutrition information determined from medical record indication of receipt of any breast milk or colostrum during the period between delivery and hospital discharge, including both mother’s own and donor breast milk (*4*). Preterm infants often have extended hospital stays (*5*); however, state statutes require completion and filing of birth certificates soon after delivery, usually within 5–10 days of birth. Therefore, among preterm infants, this item likely captures receipt of breast milk only between delivery and completion of the birth certificate. Gestational age was ascertained from the birth certificate’s obstetric estimate of completed weeks of gestation and categorized as extremely preterm (20–27 weeks), early preterm (28–33 weeks), late preterm (34–36 weeks), and term (≥37 weeks)^†^ (*4*). On birth certificates, maternal sociodemographic data are typically collected through maternal self-report and neonatal intensive care unit (NICU) admission is collected from the medical record (*4*).

Analysis was restricted to infants with gestational ages ≥20 weeks who were not transferred to another facility within 24 hours of delivery and who were living at the time of birth certificate completion. Only births delivered to residents of 48 states and the District of Columbia in 2017 were included; births delivered to residents of California and Michigan were not available for analysis (15.1% of U.S. resident births). The percentage of infants who received breast milk was calculated overall and by gestational age using SAS (version 9.4; SAS Institute). Receipt of breast milk was further stratified by maternal characteristics and infant NICU admission.

Overall, 83.9% of infants received breast milk during the first few days of life (Table). Term infants were more likely to have received breast milk than were preterm infants, with percentages increasing with gestational age: 71.3% of extremely preterm infants, 76.0% of early preterm infants, 77.3% of late preterm infants, and 84.6% of term infants.

**TABLE:** Number of infants receiving breast milk among live infants not transferred to another facility,* by gestational age^†^ and maternal and infant characteristics — 48 states^§^ and the District of Columbia, National Vital Statistics System, 2017

Characteristic	Receipt of breast milk by gestational age								Overall	
	Extremely preterm		Early preterm		Late preterm		Term			
**Total no.^¶^**	**No. received (%)**	**Total no.^¶^**	**No. received (%)**	**Total no.^¶^**	**No. received (%)**	**Total no.^¶^**	**No. received (%)**	**Total no.^¶^**	**No. received (%)**
**Overall**	13,225	9,433(71.3)	60,385	45,883 (76.0)	225,279	174,084 (77.3)	2,895,984	2,451,442 (84.6)	3,194,873	2,680,842 (83.9)
Maternal race/ethnicity**										
Hispanic	2,704	1,981 (73.3)	12,014	9,588 (79.8)	46,371	38,551 (83.1)	597,800	528,165 (88.4)	658,889	578,285 (87.8)
White	4,820	3,557 (73.8)	28,131	21,913 (77.9)	114,473	89,810 (78.5)	1,599,178	1,371,958 (85.8)	1,746,602	1,487,238 (85.2)
Black	4,620	3,102 (67.1)	15,198	10,455 (68.8)	44,636	29,580 (66.3)	436,677	320,136 (73.3)	501,131	363,273 (72.5)
Asian	545	415 (76.1)	2,700	2,217 (82.1)	10,886	9,553 (87.8)	155,841	143,134 (91.8)	169,972	155,319 (91.4)
American Indian/Alaska Native	107	65 (60.7)	527	360 (68.3)	2,268	1,525 (67.2)	24,199	18,739 (77.4)	27,101	20,689 (76.3)
Native Hawaiian/Other Pacific Islander	20	15 (75.0)	147	118 (80.3)	587	444 (75.6)	6,586	5,510 (83.7)	7,340	6,087 (82.9)
Multiracial	322	243 (75.5)	1,257	956 (76.1)	4,864	3,759(77.3)	60,134	50,523 (84.0)	66,577	55,481 (83.3)
**Maternal age group (yrs)**										
≤19	902	622 (69.0)	3,227	2,334 (72.3)	11,649	8,122 (69.7)	150,865	111,202 (73.7)	166,643	122,280 (73.4)
20–29	6,325	4,520 (71.5)	27,406	20,653 (75.4)	105,814	79,487 (75.1)	1,447,576	1,192,173 (82.4)	1,587,121	1,296,833 (81.7)
30–39	5,421	3,882 (71.6)	26,716	20,563 (77.0)	97,796	78,407 (80.2)	1,214,657	1,075,184 (88.5)	1,344,590	1,178,036 (87.6)
≥40	577	409 (70.9)	3,036	2,333 (76.8)	10,020	8,068 (80.5)	82,886	72,883 (87.9)	96,519	83,693 (86.7)
**Maternal highest education level**										
Less than a high school diploma	1,929	1,250 (64.8)	8,891	5,987 (67.3)	32,739	21,455 (65.5)	375,711	277,369 (73.8)	419,270	306,061 (73.0)
High school diploma	3,936	2,716 (69.0)	16,562	11,734 (70.8)	60,428	42,069 (69.6)	728,084	557,575 (76.6)	809,010	614,094 (75.9)
Some college	4,177	3,048 (73.0)	18,037	14,114 (78.3)	66,933	53,016 (79.2)	825,306	707,817 (85.8)	914,453	777,995 (85.1)
College graduate	3,035	2,338 (77.0)	16,229	13,623 (83.9)	63,359	56,336 (88.9)	947,414	893,004 (94.3)	1,030,037	965,301 (93.7)
**Maternal marital status**										
Married	6,044	4,571 (75.6)	31,440	25,806 (82.1)	125,837	106,251 (84.4)	1,758,860	1,593,226 (90.6)	1,922,181	1,729,854 (90.0)
Unmarried	7,181	4,862(67.7)	28,945	20,077 (69.4)	99,442	67,833 (68.2)	1,137,124	858,216 (75.5)	1,272,692	950,988 (74.7)
**Payment source at delivery**										
Medicaid	6,665	4,484 (67.3)	28,806	20,184 (70.1)	105,068	72,518 (69.0)	1,220,031	935,374 (76.7)	1,360,570	1,032,560 (75.9)
Private insurance	5,371	4,100 (76.3)	26,561	21,868 (82.3)	102,270	87,359 (85.4)	1,426,621	1,295,629 (90.8)	1,560,823	1,408,956 (90.3)
Self-pay	580	385 (66.4)	2,260	1,618 (71.6)	8,122	6,158 (75.8)	121,236	107,838 (88.9)	132,198	115,999 (87.7)
Other	505	398 (78.8)	2,374	1,936 (81.6)	8,445	7,037 (83.3)	111,100	98,552 (88.7)	122,424	107,923 (88.2)
**WIC participation during pregnancy**										
Yes	4,919	3,409 (69.3)	22,841	16,644 (72.9)	88,406	62,876 (71.1)	1,057,343	815,648 (77.1)	1,173,509	898,577 (76.6)
No	8,106	5,896 (72.7)	36,716	28,681 (78.1)	134,117	109,275 (81.5)	1,805,233	1,608,321 (89.1)	1,984,172	1,752,173 (88.3)
**Infant NICU admission**										
Yes	12,188	8,802 (72.2)	53,277	40,482 (76.0)	80,465	61,245 (76.1)	120,583	94,370 (78.3)	266,513	204,899 (76.9)
No	1,036	630 (60.8)	7,091	5,391 (76.0)	144,675	112,742 (77.9)	2,773,425	2,355,451 (84.9)	2,926,227	2,474,214 (84.6)

**Abbreviations:** NICU = neonatal intensive care unit; WIC = Special Supplemental Nutrition Program for Women, Infants, and Children.

* Excludes infants transferred to another facility within 24 hours of delivery and those who died before completion of the birth certificate.

^†^ Extremely preterm: 20–27 weeks’ gestation; early preterm: 28–33 weeks’ gestation; late preterm: 34–36 weeks’ gestation; term: ≥37 weeks’ gestation.

^§^ Includes all states except California and Michigan.

^¶^ Denominators might not sum to total because of missing maternal or infant data.

** All racial/ethnic groups are non-Hispanic unless otherwise specified.

Among extremely preterm infants, 67.1% of those delivered to black mothers and 60.7% of those delivered to American Indian/Alaska Native mothers received breast milk, compared with approximately 75% of extremely preterm infants delivered to mothers of other racial/ethnic groups. This racial/ethnic disparity was observed across gestational age groups. In general, across gestational age groups, infants of mothers who were younger, less educated, unmarried, and participating in Medicaid or the Special Supplemental Nutrition Program for Women, Infants, and Children (WIC) were less likely to receive breast milk than were infants of older, more educated, and married mothers, those with private insurance, other coverage, or who were self-pay, and those not participating in WIC. In addition, receipt of breast milk by NICU admission differed by gestational age, with higher prevalences of receipt of breast milk among late preterm and term infants who were not admitted to a NICU.

## Discussion

Although breast milk is especially beneficial for preterm infants, fewer preterm than term infants received breast milk in the first few days of life. Disparities in receipt of breast milk by gestational age could be explained by multiple factors. Gastrointestinal tract or oral-motor immaturity might inhibit enteral feeding (through the mouth or through a tube directly into the infant’s stomach) for some preterm infants, necessitating the use of parenteral, or intravenous, nutrition (*6*). In addition, mothers of preterm infants might be unable to produce sufficient breast milk and might lack access to donor milk. The American Academy of Pediatrics recommends that all preterm infants receive breast milk; if mother’s milk is unavailable or contraindicated, then fortified pasteurized donor milk should be used (*2*). Data from CDC’s 2015 Maternity Practices in Infant Nutrition and Care survey indicate that among U.S. hospitals with level 3 and level 4 NICUs, approximately 66% and 73%, respectively, report using any donor milk (*7*).

Multiple demographic factors are known to be associated with breastfeeding, including maternal age, race/ethnicity, education, and marital status.^§^ This analysis determined that many of these demographic predictors of breastfeeding are consistent across gestational ages. Infants delivered to black and American Indian/Alaska Native mothers are more likely to be born at earlier gestational ages (*1*) and are less likely to receive any breast milk. Together, these factors place these infants at increased risk for morbidity and mortality (*2*,*8*).^¶^^,^**

Hospitals and health care providers have the opportunity to improve infant nutrition. Substantial evidence has demonstrated that use of maternity care practices supportive of breastfeeding have resulted in increased breastfeeding initiation, duration, and exclusivity among term infants (*3*). Mothers of preterm infants will likely need additional support to establish and maintain a milk supply (*9*). Hospitals and health care providers can implement evidence-based policies and practices to ensure that all mother-infant dyads receive support for breast milk feeding (*3*). Prenatal breastfeeding education delivered consistently throughout the entire prenatal period might help ensure that all mothers, even those who deliver prematurely, are prepared to breastfeed or pump breast milk (*3*). In addition, hospitals can support increased access to donor milk for mothers of preterm infants, if needed and desired, to help preterm infants receive breast milk as soon as receipt is medically feasible (*2*). Finally, to address the challenges that caregivers could encounter when feeding infants hospitalized for extended periods, hospitals might also consider providing support such as helping mothers prepare for long-term breast milk pumping and providing follow-up lactation consultations throughout an infant’s hospitalization.

Quality improvement initiatives, such as CDC-supported state-based perinatal quality collaboratives,^††^ seek to rapidly implement these best practices in hospitals and work to increase use of human milk in the neonatal intensive care setting and improve support for breastfeeding in hospitals and in the community. Increased implementation of similar initiatives in hospitals serving larger proportions of racial/ethnic groups with lower breast milk feeding rates might help to decrease disparities in breast milk feeding and improve infant morbidity and mortality.

CDC’s National Immunization Survey is used for routine surveillance of breastfeeding initiation, duration, and exclusivity; however, this data source does not include gestational age. Overall rates of receipt of breast milk calculated from 2017 birth certificate data are comparable to breastfeeding initiation rates estimated from the survey data (83.2% among infants born in 2015).^§§^

The findings in this report are subject to at least three limitations. First, birth certificate data do not allow for analysis of breast milk feeding duration or exclusivity, which are important indicators of optimal infant feeding practices. Second, because an infant’s birth certificate might be completed before enteral nutrition is medically feasible, birth certificate data might not capture properly delayed introduction of breast milk among preterm or medically fragile infants. Finally, although analysis was restricted to infants not transferred to another facility, some variables might be misclassified. A comparison of birth certificate data with medical records in eight hospitals across two states found high exact agreement for obstetric estimate of gestation within 2 weeks (99.7% and 98.1% in each state) and high sensitivity for receipt of breast milk (90.7% and 96.2%). However, moderate false discovery rates for receipt of breast milk (the percentage of births with birth certificate but not medical record indication) (19% and 16% for each of the two states) suggest there might be discrepancies between medical records and birth certificate reporting in some hospitals (*10*). In addition, rates of breast milk feeding among extremely and early preterm infants not admitted to the NICU should be interpreted with caution. These infants likely required advanced medical care but might have been misclassified as non-NICU admissions because of incorrect birth certificate data or NICU admission after completion of the birth certificate.

Infants’ receipt of breast milk as soon as is medically feasible can help prevent infection and promote growth and development. Receipt of breast milk is important for preterm infants because breast milk also helps protect against necrotizing enterocolitis (*2*), an important contributor to gastrointestinal morbidity and mortality among preterm infants. Hospital enactment and provision of evidence-based policies and practices that support breast milk feeding and donor milk access for all infants at high risk (*2*,*3*), as well as development of infant feeding policies and practices that promote breast milk feeding among mother-infant dyads facing challenges associated with extended infant hospitalizations, could help reduce gestational age disparities in the receipt of breast milk and increase the proportion of all infants receiving the benefits of breast milk.

**Summary**

**What is already known about this topic?**

Breast milk is the optimal source of infant nutrition. Data on breast milk intake by gestational age are limited.

**What is added by this report?**

Rates of receipt of breast milk among extremely preterm, early preterm, late preterm, and term infants were 71.3%, 76.0%, 77.3%, and 84.6%, respectively, among infants delivered to residents of 48 states and the District of Columbia in 2017.

**What are the implications for public health practice?**

Disparities in receipt of breast milk by gestational age exist. Hospital implementation of policies and practices that ensure that all mothers and their infants receive support for breast milk feeding and that preterm infants receive breast milk as soon as is medically feasible might help reduce these disparities.

## Footnotes

* https://www.cdc.gov/nchs/data/dvs/birth11-03final-ACC.pdf.

† Gestational age terms are those commonly used by various medical, research, and public health organizations. However, because there is a lack of consensus regarding the age ranges for each category, categories were defined in this report using completed weeks’ gestation as the following: extremely preterm (20–27 weeks), early preterm (28–33 weeks), late preterm (34–36 weeks), and term (≥37 weeks). The lower limit of 20 completed weeks’ gestation was used to exclude births where resuscitation was unlikely.

§ https://www.cdc.gov/breastfeeding/data/nis_data/rates-any-exclusive-bf-socio-dem-2015.htm.

¶ https://www.cdc.gov/reproductivehealth/maternalinfanthealth/pretermbirth.htm.

** https://www.cdc.gov/reproductivehealth/maternalinfanthealth/infantmortality.htm.

†† https://www.cdc.gov/reproductivehealth/maternalinfanthealth/pqc.htm.

§§ https://www.cdc.gov/breastfeeding/data/nis_data/results.html.
